# Intentional Weight Loss and All-Cause Mortality: A Meta-Analysis of Randomized Clinical Trials

**DOI:** 10.1371/journal.pone.0121993

**Published:** 2015-03-20

**Authors:** Stephen B. Kritchevsky, Kristen M. Beavers, Michael E. Miller, M. Kyla Shea, Denise K. Houston, Dalane W. Kitzman, Barbara J. Nicklas

**Affiliations:** 1 Department of Internal Medicine, Wake Forest School of Medicine, Winston-Salem, NC, United States of America; 2 Department of Health and Exercise Science, Wake Forest University, Winston-Salem, NC, United States of America; 3 Department of Biostatistical Sciences, Wake Forest School of Medicine, Winston-Salem, NC, United States of America; 4 Human Nutrition Research Center on Aging, Tufts University, Boston, MA, United States of America; Providence VA Medical Center and Brown University, UNITED STATES

## Abstract

**Background:**

Obesity is associated with increased mortality, and weight loss trials show rapid improvement in many mortality risk factors. Yet, observational studies typically associate weight loss with higher mortality risk. The purpose of this meta-analysis of randomized controlled trials (RCTs) of weight loss was to clarify the effects of intentional weight loss on mortality.

**Methods:**

2,484 abstracts were identified and reviewed in PUBMED, yielding 15 RCTs reporting (1) randomization to weight loss or non-weight loss arms, (2) duration of ≥18 months, and (3) deaths by intervention arm. Weight loss interventions were all lifestyle-based. Relative risks (RR) and 95% confidence intervals (95% CI) were estimated for each trial. For trials reporting at least one death (n = 12), a summary estimate was calculated using the Mantel-Haenszel method. Sensitivity analysis using sparse data methods included remaining trials.

**Results:**

Trials enrolled 17,186 participants (53% female, mean age at randomization = 52 years). Mean body mass indices ranged from 30–46 kg/m^2^, follow-up times ranged from 18 months to 12.6 years (mean: 27 months), and average weight loss in reported trials was 5.5±4.0 kg. A total of 264 deaths were reported in weight loss groups and 310 in non-weight loss groups. The weight loss groups experienced a 15% lower all-cause mortality risk (RR = 0.85; 95% CI: 0.73–1.00). There was no evidence for heterogeneity of effect (Cochran’s Q = 5.59 (11 d.f.; p = 0.90); I^2^ = 0). Results were similar in trials with a mean age at randomization ≥55 years (RR = 0.84; 95% CI 0.71–0.99) and a follow-up time of ≥4 years (RR = 0.85; 95% CI 0.72–1.00).

**Conclusions:**

In obese adults, intentional weight loss may be associated with approximately a 15% reduction in all-cause mortality.

## Introduction

Advanced age and obesity are risk factors for disability, morbidity, and mortality [1–3]. Weight loss interventions in overweight and obese older adults positively affect several strong risk factors for mortality, including: circulating IL-6 levels [4–6], blood pressure [7,8], fasting plasma glucose [9,10], gait speed [11–13], and cardiorespiratory fitness [12,14,15]. Yet, many observational studies in middle-aged and older adults report an association between weight loss and increased mortality [16–18]. Difficulty reconciling these contradictory findings (the so-called “obesity paradox”), coupled with the strong negative prognostic implication of rapid involuntary weight loss with advanced age, has led to a reluctance to recommend weight loss in older adults [19].

Attempts to refine observational analyses to avoid confounding (i.e. distinguishing between intentional and unintentional weight loss, and restricting populations to those without co-morbid conditions or non-smokers) typically reveal no increase, and perhaps some decrease, in mortality risk with intentional weight loss [20,21]. Indeed, results from the Swedish Obesity Study show a 24% reduction in all-cause 10-year mortality associated with gastric banding compared to matched-obese controls [22]. However, as a non-randomized study it is unclear if selection bias or confounding contributed to the observed mortality advantage. Although results from a randomized controlled trial (RCT) of weight loss would theoretically resolve these issues, such a trial would require a large sample size over a long duration to detect clinically meaningful differences in mortality.

In light of the high prevalence of obesity, its negative impact on health and quality of life, and the discrepancy between the proven risk factor improvements of short-term intentional weight loss and the inverse association of weight loss with increased all-cause mortality frequently seen in observational studies, we conducted a meta-analysis to estimate the effect of interventions which included intentional weight loss on all-cause mortality in overweight and obese adults. We hypothesized that intentional weight loss would be associated with reduced all-cause mortality. Further, as weight loss in older persons is a cause of clinical concern that may lead health care providers to recommend against weight loss for obese, older adults, we sought to examine the effects in a subset of trials with a mean baseline age of at least 55 years.

## Materials and Methods

### Study selection and data extraction

We sought to identify all published RCTs of intentional weight loss that reported mortality data either as an endpoint or as an adverse event, including study designs where participants were randomized to weight loss or non-weight loss, or weight loss plus a co-intervention (e.g. weight loss plus exercise) or the weight stable co-intervention (i.e. exercise alone). A comprehensive literature search was conducted using the PUBMED database (National Library of Medicine, Bethesda, MD) inclusively through December 7, 2013 on RCTs using the medical subject headings: weight loss, humans, and adult. References within identified papers were also examined for potential inclusion. Articles retrieved using this search string were then limited to trials including weight loss and non-weight loss arms, a trial duration (weight loss and maintenance phase) ≥18 months, and mortality data by intervention group.

Data were extracted in duplicate by two of the authors (SBK and KMB) and included intervention duration and length of follow up, number of participating subjects, population characteristics (age, gender, baseline BMI and health status), intervention arm descriptions, initial weight loss, and number of deaths reported. Manuscript authors were contacted for clarification when necessary.

### Statistical methods

Relative risks (RR) and 95% confidence intervals (95% CI) were estimated for each trial. Three trials reported no deaths in one of the intervention arms, thus the estimate of the RR was undefined for those trials. For the 12 trials with at least one death in each intervention group, the Mantel-Haenszel estimator of the common RR was estimated from the stratified 2x2 tables relating the intervention to mortality, with trial being the stratifying factor; 95% CIs on the common estimate were calculated using the variance estimate of Greenland and Robins (1985) [23]. Cochrane’s Q statistics and Higgins I^2^ were used to evaluate heterogeneity of the RR across trials [24]. In addition, following the recommendations of Bradburn et al. (2007) [25], sensitivity analyses were performed using the reciprocal of the number of participants in the other intervention arm as the continuity correction [26], and re-estimating the RR with the Mantel-Haenszel estimator, thus including all 15 trials when obtaining a common estimate. Lastly, three distinct sub-analyses were performed in which trials limited to those reporting: (1) relatively older participants (≥55 years of age at baseline), (2) longer follow-up periods (≥4 years), and (3) at least five kg weight loss in the weight loss intervention arm.

## Results

### Study selection and publication bias

**Fig 1:**
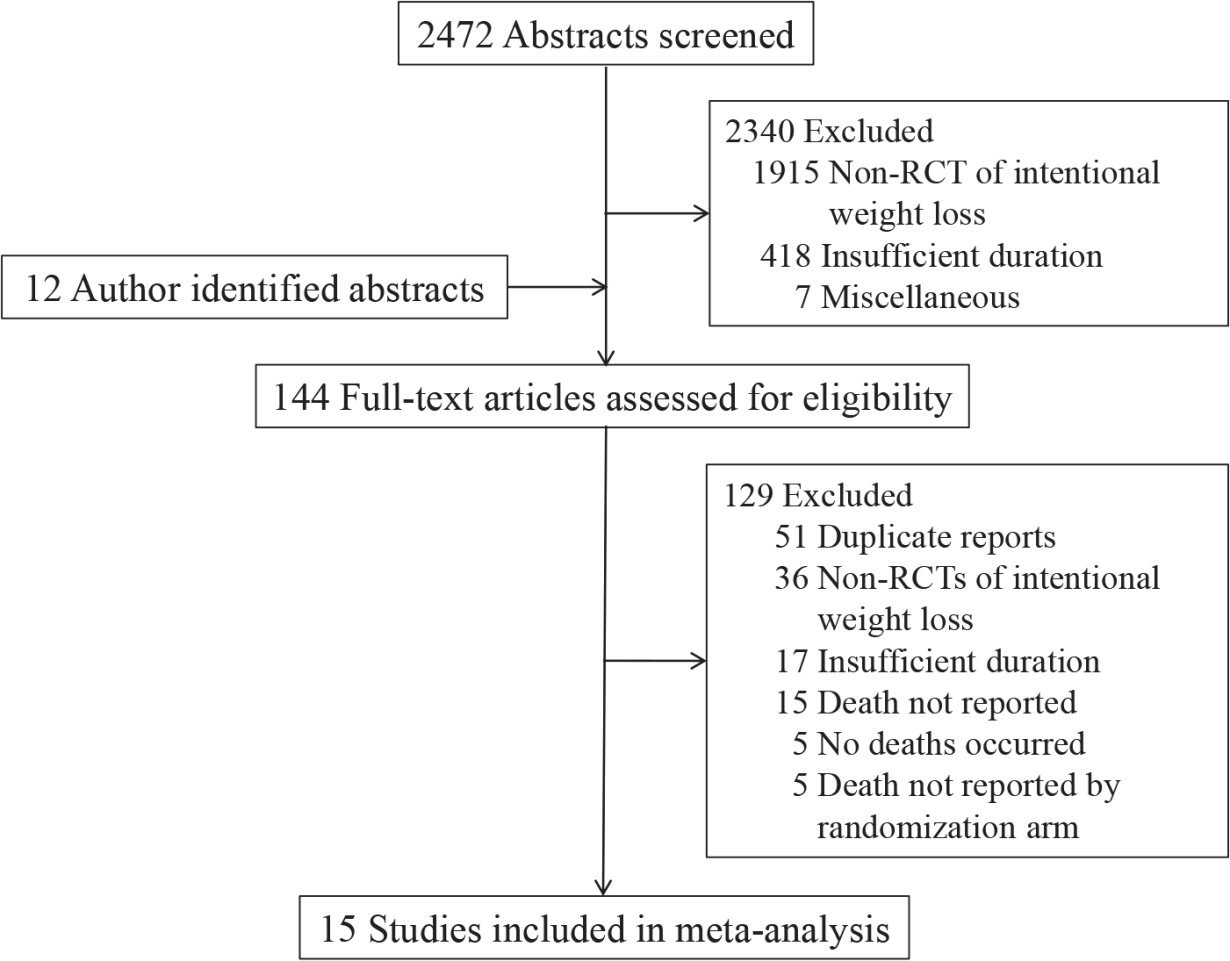
Study Selection Flowchart. Flowchart for the selection of eligible studies.

**Fig. 1** shows the study selection diagram. Our search string generated 2,472 abstracts which were initially screened for potential inclusion, of which 2,340 did not meet inclusion criteria: 1,915 were non-RCTs of intentional weight loss or compared participants receiving varied degrees of caloric restriction; 418 were of insufficient duration; and, seven were judged to be out of scope for miscellaneous reasons. Twelve abstracts came to the attention of study authors by other means (e.g. references within identified papers), yielding a total of 144 full-text articles which were independently assessed (by SBK and KMB) for eligibility. Fifty-one articles were duplicate reports, 26 were non-RCTs of intentional weight loss or compared participants receiving varied degrees of caloric restriction, 17 were of insufficient duration, 15 did not report mortality data, five reported that no deaths occurred, and in five trials death was reported, but not by randomization arm (when indicated, authors were contacted to attempt to retrieve missing information). Thus, this meta-analysis consists of data from 15 RCTs of intentional weight loss [15,27–40].

**Fig 2:**
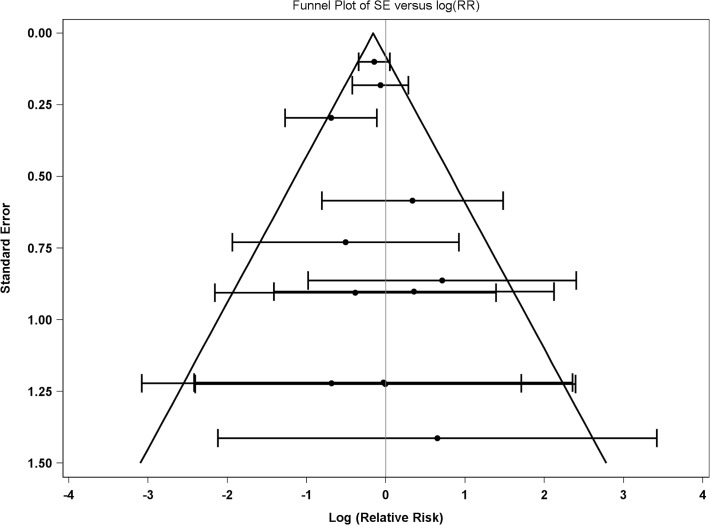
Funnel Plot. Funnel plot of the point estimate and 95% confidence interval of 12 randomized clinical trials of intentional weight loss.

Of the 15 included studies, three did not have any deaths in one of the intervention arms [32,34,35]. The remaining 12 studies were used for estimating the common relative risk using the Mantel-Haenszel approach. To assess potential publication bias, a funnel plot of the data was produced (see **Fig. 2**). The pattern does not indicate that the smaller trials were more likely to observe a result that differs from the overall result indicating a lower likelihood of publication bias.

### Study and participant characteristics

A summary of study details is presented in **Table 1**. The 15 eligible RCTs included a total of 17,186 participants (53% female) with an average age of 52 years at baseline. A total of 574 deaths were reported: 264 among those assigned to weight loss and 310 among non-weight loss comparison groups. Included trials were conducted over about 25 years, with the earliest trial published in 1987 [27]. The health status of the study-specific target populations varied and included: hypertension [27–30,36], osteoarthritis [33], pre-diabetes/diabetes [31,39], and overweight/obesity [15,32,34,35,37,38,40]. In all cases, reported mean baseline body mass index (BMI) classified participants as obese (range: 30–46 kg/m^2^), with an average BMI among trials of 35 kg/m^2^ (BMI was not reported in four trials [27–30]).

**Table 1:** Study characteristics of randomized controlled trials of weight loss interventions.

Study and Author (year)	Target Population	Intervention Arm Descriptions	Average Age (years)	Baseline Body Mass Index (kg/m^2^)	Women (%)	Length of Intervention / Maintenance*(months)	Length of Follow-Up (months)	N (# of Deaths)
HCP; Stamler (1987) [27]	Hypertensive	Weight loss w/ Na^+^ and ETOH restriction	57	—-	35	48	48	97 (3)
		Control w/o pharmacotherapy	55		38			92 (2)
TOHP I (1992) [29]	Hypertensive	Weight loss	43	—-	27	18	18	308 (1)
		Control	43		37			1158 (1)
TAIM; Davis (1993) [28]	Overweight/obese hypertensive	Weight loss w and w/o pharmacotherapy	48	—-	46	30	54	291 (4)
		Usual diet w/ and w/o pharmacotherapy						296 (2)
TOHP II (1997) [30]	Overweight/obese hypertensive	Weight loss w/ and w/o Na^+^ restriction	44	—-	34	6/30	36	1192 (7)
		No weight loss w/ and w/o Na^+^ restriction			33			1190 (5)
DPP; Knowler (2002) [31]	Pre-diabetic	Dietary weight loss and exercise	51	34	68	34	34	1079 (3)
		Placebo tablets			69			1082 (5)
Johnson (2008) [32]	Overweight/obese	Multiple behavioral change weight loss	45	31	46	9	24	628 (0)
		Control	45	31	49			649 (3)
ADAPT; Shea (2010) [33]	Osteoarthritic	Dietary weight loss w/ and w/o exercise	69	34	72	6/12	96	159 (15)
		Exercise and attention control			71			159 (30)
LOSS; Ryan (2010) [34]	Morbidly obese	Diet, behavior, medication therapy	47	46	84	24	24	200 (1)
		Usual care	47	47	84			190 (0)
ORBIT; Fitzgibbon (2010) [35]	Obese	Culturally proficient weight loss	46	39	100	6/12	18	107 (1)
		Control	46	39	100			106 (0)
TONE; Shea (2011) [36]	Hypertensive	Weight loss w/ and w/o Na^+^ restriction	66	31	47	8/22	152	294 (49)
		Na+ restriction or attention control			57			291 (52)
WOMAN; Gabriel (2011) [37]	Overweight/Obese	Weight loss and exercise	57	31	100	36	48	253 (1)
		Health education control	57	31	100			255 (2)
CLIP; Rejeski (2011) [15]	Overweight/obese w/CVD	Weight loss and exercise	67	33	68	6/12	18	98 (1)
		Exercise or successful aging	67	33	66			190 (2)
ALIFE@Work; van Wier (2011) [38]	Overweight	Phone/internet delivered weight loss	43	30	33	6	24	926 (2)
		Control	43	30	33			460 (1)
Look AHEAD; Wing (2013) [39]	Type 2 diabetic	Dietary weight loss and exercise	59	36	59	12/103	115	2570 (174)
		Attention control			60			2575 (202)
ACHIEVE; Daumit (2013) [40]	Overweight/obesew/mental illness	Dietary weight loss and exercise	47	36	51	18	18	144 (2)
		Control	44	37	49			147 (3)

*Intervention duration refers to the total time period in which weight loss was advocated (not including weight loss maintenance).

Weight loss interventions were all lifestyle-based, with an average duration of 27 months (range: six-96 months). Only three trials considered mortality as an endpoint [33,36,39]; other trials reported death as an adverse event. A weight loss goal of 5–10% of baseline weight was specified in nine trials [15,27,28,30,31,33,35,36,39]. For trials that reported average initial weight loss (n = 6), the average initial weight loss in the weight loss and non-weight loss arms was 5.5 kg (range: -1.8 to -13.1 kg) and 0.2 kg (range: -1.1 to +0.2), respectively [29,33–36,40].

### Weight loss and mortality

**Fig 3:**
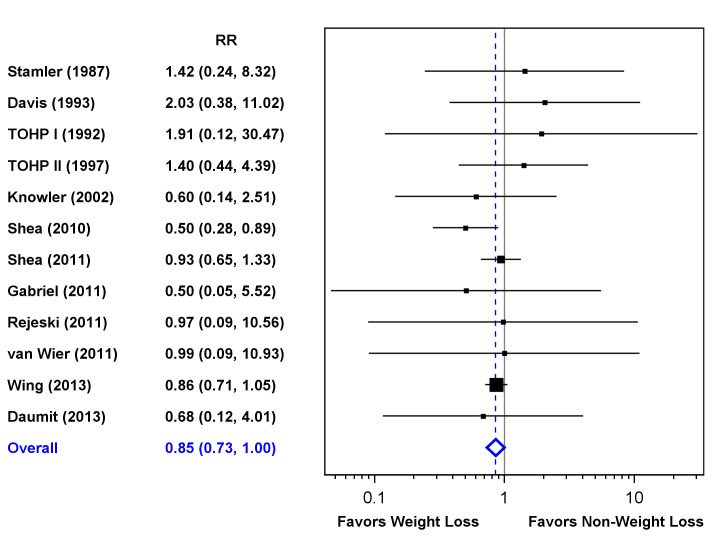
Forest Plot. Forest plot showing individual and pooled relative risks of all-cause mortality with 95% confidence intervals across 12 randomized clinical trials of weight loss interventions. Three trials did not report deaths in one intervention arm and are not included in this figure.

**Fig. 3** shows the point estimates, 95% CIs, and summary RRs for mortality for the 12 trials that reported deaths in each arm (death n = 569). Across all trials, there was a 15% reduction in all-cause mortality for participants randomized to weight loss (RR = 0.85; 95% CI: 0.73–1.00). Cochran’s Q was 5.59 (11 d.f.; p = 0.90), with an associated I^2^ = 0, indicating no evidence of heterogeneity of the RR among the individual trials. Inclusion of the three trials with sparse data, after applying continuity corrections, resulted in an identical estimate (RR = 0.85; 95% CI 0.73–1.00). Six of the 15 trials had point estimates favoring weight loss, and ADAPT [33] showed a significant benefit for weight loss. Only three trials (ADAPT [33], TONE [36], Look AHEAD [39]) contributed more than 30 deaths to the analysis, with Look AHEAD contributing 65.5% of the total deaths. Total mortality was lower in the weight loss arms in each of these trials. The summary estimate omitting Look AHEAD data was 0.83 (95% CI 0.64–1.08).

### Sub-analyses: modifying effects of age, follow up duration, and magnitude of weight loss

Six trials had a mean age at randomization ≥55 years [15,27,33,36,37,39]. The summary estimate for these trials was 0.84 (95% CI 0.71–0.99). Six trials reported follow-up times ≥4 years [27,28,33,36,37,39]; the summary estimate for these trials was 0.85 (95% CI 0.72–1.00). Not all trials reported the degree of weight loss achieved. In the six trials in which an average weight loss of at least five kg was reported for the weight loss intervention arm [15,29,31,36,37,39], the summary estimate was 0.88 (95% CI: 0.74–1.04).

## Discussion

In this meta-analysis of 15 RCTs of intentional weight loss in obese adults, the risk of all-cause mortality was 15% lower for individuals randomized to weight loss, compared to non-weight loss, groups. Results did not materially differ when examining only the trials of relatively older participants, trials with longer follow-up periods, or those reporting at least five kilograms of weight loss.

Although we present novel summary data on the effects of intentional weight loss on mortality risk from RCTs, our findings are comparable to a 2009 meta-analysis of the effect of lifestyle-based weight loss on all-cause mortality risk from prospective studies [20]. In this analysis of 26 studies, authors concluded that unintentional, but not intentional, weight loss increases risk of mortality. Akin to our results, authors also report intentional weight loss reduces all-cause mortality risk by approximately 15% in individuals with obesity-related risk factors; however, this finding did not extend to overweight (but not obese) or otherwise healthy individuals. Despite the overall finding, it is worth noting that several of the observational studies included in this meta-analysis found self-reported intentional weight loss to be associated with increased risk of mortality [20]. It is possible that the attribution of intentionality is unreliable, or that in some persons unintentional and intentional weight loss occur simultaneously. RCT data presented here circumvent potential confounding by self-report in observational studies; importantly, we found an upper boundary of the 95% CI for the association between randomization to weight loss and mortality of 1.00, thus providing evidence that a mortality excess may not exist in obese adults who lose weight intentionally.

Undertaking of this meta-analysis was partly motivated by the desire to resolve uncertainty regarding the long-term safety of weight loss for older adults. In addition to mortality, theoretical long-term safety concerns relate to the loss of muscle and bone mass that occur with weight loss [41], which might predispose older adults to impaired physical function and increased fracture risk. The mortality point estimate for the six trials with a mean age ≥55 years at baseline did not differ from the overall estimate; however, only three trials specifically limited their target population to older adults (i.e. mean baseline age of >65 years). Of these, ADAPT [33] showed a statistically significant benefit of weight loss, TONE [36] tended to favor weight loss, and CLIP [15] showed no effect. While these results are reassuring for geriatricians contemplating the recommendation of weight loss to their obese patients, more long-term data is needed to better understand the net benefits and risks of intentional weight loss in this population.

The most straightforward mechanism by which weight loss might reduce mortality in overweight and obese older adults is through the improvement of risk factors that either predict mortality on their own, or contribute to overall mortality through obesity-related disease (e.g. stroke and heart disease). The data needed to assess mediation by these factors was not available. There are clear benefits of weight loss for the reduction of strong mortality risk factors in older adults including increased peak VO_2_ [12] and walking speed [5,13,15], and reduced circulating IL-6 [5,6], blood pressure [8], and glucose levels [10]. Uncertainty exists, however, over what length of time the effects of weight loss on mortality might manifest themselves. Results from the Swedish Obesity Study [22]and the Look AHEAD trial [39] suggest mortality benefit only appears after four to five years of follow-up. However, data from the 18-month ADAPT trial showed apparent benefit over the entire course of the post-trial follow-up [33]. We excluded studies lasting fewer than 18 months because deaths occurring within a short-time after randomization are more likely due to pathological processes active at randomization rather than the intervention itself. Only three trials (HCP [27], WOMAN [37], and Look AHEAD [39]) were designed with an intervention length greater than 36 months, making it difficult to reach a conclusion with respect to intervention duration; however, restricting the meta-analysis to the six studies with at least four years of follow-up time gave similar results as the overall analysis. Lastly, several studies show that weight gain, especially in persons who are already obese, is a strong risk factor for mortality [42–44]. Thus, the protective effect of weight loss on mortality may relate to interrupting this trajectory.

This meta-analysis has several limitations. First, as with all meta-analyses, our results depend on the quality and consistency of data presented in the source documents. Data presentation styles were inconsistent and affected by changing reporting practices over time. For example, BMI was unreported in four of the earliest trials and weight loss targets/end of trial weights were reported as absolute amounts or percentages, or were unreported in several trials. Second, we were not able to include five trials which did not consider (or report) deaths by intervention arm. These trials tended to be smaller, in relatively younger populations, and of short-duration; thus, the impact of these missing trials on the overall effect measure is likely to be small. Third, our inclusion criteria were heterogeneous with trials targeting persons with hypertension, diabetes or osteoarthritis. Data are too sparse to conclude that the benefits of weight loss relate to any specific baseline condition. No cause of death information was identified, so we cannot comment on whether the observed mortality benefit is due to the reduction of specific causes. Fourth, in many trials, persons in the non-weight loss arms received active interventions, including in some cases pharmacotherapy or exercise training. It is possible that these interventions may have had an effect on mortality in the comparison groups and whether this would tend to magnify or diminish the group mortality differences is unclear. Additionally, the vast majority of weight loss arms coupled caloric restriction with an additional therapy (i.e. sodium restriction, exercise training). Thus, it is reasonable to speculate whether the observed mortality benefit of “weight loss” is attributable to weight loss alone. Although limited data exist to answer this question, results from the ADAPT study [33], where participants were randomized to weight loss and long-term exercise, alone or in combination, attribute the observed mortality benefit to weight loss (rather than exercise). Fifth, the degree to which different health behaviors adopted during the active intervention phase may have been maintained after the end of the trial is unclear, and there are no data on the extent to which weight-related mortality risk factors may have changed after the conclusion of any of the trials. Lastly, because our study selection criteria deliberately excluded trials in which randomized groups received differing degrees of weight loss, we cannot comment on a weight loss/mortality dose-response. Although, when trials were restricted to those reporting the greatest weight reductions (i.e. >5 kg), results differed little from the overall effect estimate.

## Conclusion

In conclusion, this meta-analysis of 15 randomized controlled trials of weight loss in obese and overweight adults shows a 15% reduction in all-cause mortality in those randomized to weight loss. The magnitude of this benefit is on par with the reductions in all-cause mortality risk seen with treating hypertension or reducing total serum cholesterol by 1 mmol/L [45,46]. Most of the relevant literature in this area pertains to middle-aged adults. Given the increasing prevalence of obesity in older adults and its impact on physical function and chronic disease, additional evidence from well-conducted trials in older adults is needed to clarify the long-term safety of intentional weight loss in this population [47].

## Supporting Information

S1 PRISMA Checklist Completed PRISMA (2009) checklist pertaining to the content of this meta-analysis. (PDF)
